# An Association Analysis between Mitochondrial DNA A10398G Polymorphism and Temperament in Japanese Young Adults

**DOI:** 10.1371/journal.pone.0007763

**Published:** 2009-11-09

**Authors:** Kunihiro Kishida, Mihoko Tominaga, Kiminori Matsubara, Masanori Taguchi, Masanori Noguchi, Noriaki Tsunawake, Yoshihiro Shidoji

**Affiliations:** 1 Graduate School of Human Health Sciences, University of Nagasaki, Siebold, Nagayo, Nagasaki, Japan; 2 Graduate School of Education, Hiroshima University, Higashi-Hiroshima, Hiroshima, Japan; 3 Department of Language and Culture, Dokkyo University, Soka-shi, Saitama, Japan; 4 Department of General Education, Sasebo National College of Technology, Sasebo, Nagasaki, Japan; RIKEN Brain Science Institution, Japan

## Abstract

The mitochondrial (mt) DNA C5178A and A10398G polymorphisms have been reported to be associated with mental disorders such as bipolar disorder. However, the effects of these polymorphisms on temperament in healthy people are poorly understood. Evaluating healthy subjects can have the advantage of providing new strategies for maintaining psychological health and preventing mental illness. We examined the association between mtDNA polymorphisms and temperament in Japanese students. There was no significant difference in examined temperament when analysed by genotypes, 5178–10398 haplotypes, or sex. The subgroup analysis based on sex indicated that there was an interactive effect of the mtDNA A10398G polymorphism and sex on anxiety and obsession. This finding is preliminary and cannot exclude the possibility of false-positive due to small sample size (144 subjects) and multiple statistical testing. Further studies involving a larger sample size or other ethnic groups are necessary to confirm that mtDNA A10398G polymorphism can be a genetic factor for temperament.

## Introduction

Mitochondria are major organelles which generate adenosine triphosphate (ATP) for energy production. In addition to supplying cellular energy, they are involved in the regulation of calcium, which plays considerable roles in neuronal functions, such as apoptosis [1] and synaptic plasticity [2]. The mitochondrial (mt) DNA A10398G polymorphism, the missense Thr114Ala variation, is common in diverse populations and has been reported to be associated with intracellular calcium dynamics [3] and neuropsychiatric disorders, such as Parkinson disease [4] and bipolar disorder [5]. Kato et al. are proposing mitochondrial dysfunction hypothesis involving the 10398A genotype in the pathophysiology of bipolar disorder [6]. The mtDNA C5178A polymorphism, the missense Leu237Met variation, is common in almost only Asian and also has been reported to be associated with bipolar disorder [7]. However, the effects of these polymorphisms on temperament in healthy people are poorly understood. Only one report suggests the mtDNA C5178A polymorphism may be involved in personality trait [8]. Evaluating healthy subjects can have the advantage of providing new strategies for maintaining psychological health and preventing mental disorders. In this study, we controlled the possible confounding factors (i.e., aging, occupation) and examined the association between mtDNA polymorphisms and temperament in young Japanese students.

## Results and Discussion

The frequencies of the 5178C and 5178A genotypes were 63.9% and 36.1%, respectively. The frequencies of the 10398A and 10398G genotypes were 38.2% and 61.8%, respectively. Both frequencies were comparable with those in previous Japanese studies [5], [7]–[9], which validates the genotyping method used in this study. Hardy-Weinberg equilibrium tests are not valid for mtDNA polymorphisms, and therefore were not assessed, and no heteroplasmy was observed.

**Figure 1:**
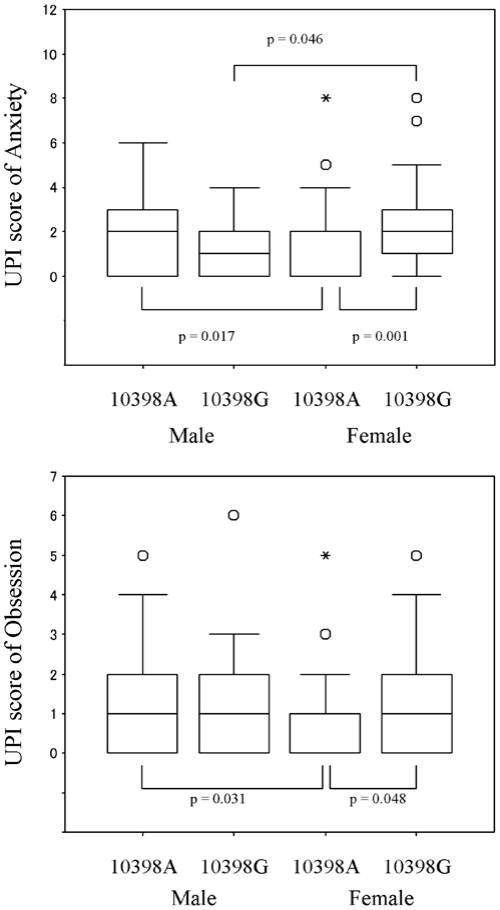
Box plot of UPI scores calculated by mitochondrial DNA A10398G genotypes and sex. The line dividing the box indicates the median value. The vertical bars indicate the range and the horizontal boundaries of the box represent the first and third quartiles. Outliers lie more than 1.5 times the interquartile range from the first or third quartile and are indicated by the presence of an open dot. Extreme outliers lie more than 3 times the interquartile range from the first or third quartiles and are indicated by the presence of an asterisk.

No significant difference in all University Personality Inventory (UPI) scores was observed when analysed by genotypes, 5178–10398 haplotypes, or sex (Table 1) (data not shown for 5178–10398 haplotypes). Although there is no prior assumption that mtDNA polymorphisms have an interaction with gender, the subgroup analysis based on sex was performed as an exploratory analysis. As a result, an interactive effect was observed between the mtDNA A10398G genotypes and sex on temperament (Table 2), while an interaction between the C5178A genotypes and sex was not found (Table 3). Table 2 describes the UPI scores calculated on the basis of A10398G genotypes and sex. In female subjects, anxiety and obsession scores were significantly higher among those with the 10398G genotype than those with the 10398A genotype, while no significant association was observed between genotypes and UPI scores in male subjects. In subjects with the 10398A genotype, anxiety and obsession scores were significantly higher in males as compared with females. In subjects with the 10398G genotype, the converse was true: anxiety scores were significantly lower in males than in females (Figure 1).

**Table 1:** UPI scores calculated on the basis of mitochondrial DNA genotypes or sex.

	C5178A Genotype		A10398G Genotype		Sex
UPI scores	5178C (n = 92)	5178A (n = 52)	10398A (n = 55)	10398G (n = 89)	Male (n = 39)	Female (n = 105)
Total	8.71±7.19	9.96±7.41	8.56±7.41	9.53±7.20	9.36±8.24	9.09±6.92
Physical complaint	2.20±2.10	2.23±2.10	2.35±2.25	2.12±1.99	2.44±2.58	2.12±1.89
Depression	3.97±3.57	4.62±3.83	4.04±3.61	4.30±3.72	3.90±3.99	4.31±3.55
Anxiety	1.63±1.85	1.79±1.71	1.45±1.86	1.83±1.75	1.67±1.72	1.70±1.83
Obsession	1.09±1.30	1.33±1.54	1.02±1.33	1.27±1.43	1.36±1.55	1.10±1.33

Values are expressed as means±S.D.s.

Higher scores indicate stronger status.

Statistical analysis was carried out using the Mann-Whitney U-test.

No significant difference was detected.

**Table 2:** UPI scores calculated on the basis of mitochondrial DNA A10398G genotypes and sex.

	Male		Female	
UPI scores	10398A (n = 18)	10398G (n = 21)	10398A (n = 37)	10398G (n = 68)
Total	11.28±9.52	7.71±6.76	7.24±5.84	10.09±7.28
Physical complaint	2.83±2.79	2.10±2.40	2.11±1.94	2.13±1.87
Depression	4.61±4.62	3.29±3.36	3.76±3.02	4.62±3.78
Anxiety	2.22±1.95*	1.19±1.36†	1.08±1.72††	2.03±1.81
Obsession	1.61±1.65*	1.14±1.45	0.73±1.04†	1.31±1.42

Values are expressed as means±S.D.s.

Higher scores indicate stronger status.

Statistical analysis was carried out using the Mann-Whitney U-test.

* P<0.05 vs. female with 10398A.

† P<0.05 vs. female with 10398G.

†† P<0.01 vs. female with 10398G.

**Table 3:** UPI scores calculated on the basis of mitochondrial DNA C5178A genotypes and sex.

	Male		Female	
UPI scores	5178C (n = 28)	5178A (n = 11)	5178C (n = 64)	5178A (n = 41)
Total	10.00±8.30	7.73±8.25	8.14±6.65	10.56±7.16
Physical complaint	2.75±2.65	1.64±2.34	1.95±1.79	2.39±2.04
Depression	4.07±4.06	3.45±3.98	3.92±3.37	4.93±3.78
Anxiety	1.79±1.80	1.36±1.57	1.56±1.88	1.90±1.74
Obsession	1.39±1.45	1.27±1.85	0.95±1.21	1.34±1.48

Values are expressed as means±S.D.s.

Higher scores indicate stronger status.

Statistical analysis was carried out using the Mann-Whitney U-test.

No significant difference was detected.

The mtDNA A10398G polymorphism results in a nonsynonymous amino acid substitution from threonine (A allele) (hydrophilic) to alanine (G allele) (hydrophobic) within the nicotinamide adenine dinucleotide (NADH) dehydrogenase subunit of complex I of the electron transport chain. The 10398G genotype characterizes European haplogroup I, J, K and Asian-specific super haplogroup M, and is more frequent than the 10398A genotype in Asians, whereas the frequency of G allele is smaller in Europeans [10], [11]. This change has been reported to alter mitochondrial matrix pH and calcium dynamics [3], which might affect neuroplasticity and neural transmission with subsequent effects on temperament. The 10398A genotype has been reported to be associated with bipolar disorder, which is thought to be due to the above altered calcium dynamics, that is, calcium dysregulation [3]. In this study, the 10398G genotype was associated with anxiety and obsession in female subjects despite the result of subdivision analysis. The discrepancy between genotypes or genders may arise from the following reasons. As UPI test is a simple screening test to evaluate the mental state of university students, slightly higher UPI scores in healthy subjects do not always reflect a preclinical stage of mental disease. The subjects recruited in the present study had the comparatively homogeneous backgrounds: ethnically Japanese; university students; and small difference in age, which has advantages in statistical analyses. It should be noted, however, that the observed results might be limited to the foregoing backgrounds. Additionally, this study involved small sample size and multiple statistical testing, which is liable to a false-positive result. These limitations suggest that this finding is preliminary and requires further researches.

The only previous study examining personality traits in healthy volunteers [37.8±12.0 years of age (mean±SD)] reported an association between the 5178A genotype, but not the A10398G, and extraversion in both sexes [8], which seems inconsistent with our findings. However, our data were obtained from students whose age and occupation were different from the sample used in the previous study. In addition, differences in examined temperament might have resulted in differences in outcome.

In the present study, we observed an interactive effect of the mtDNA A10398G polymorphism and sex on anxiety and obsession. This finding is marginal and can not rule out the possibility of false-positive as described above. Further studies involving a larger sample size or other ethnic groups will be necessary to confirm that mtDNA A10398G polymorphism can be a genetic factor for temperament.

## Methods

### Ethics Statement

The study was performed in accordance with the Declaration of Helsinki and approved by the Research Ethics Committee of the University of Nagasaki and Hiroshima University. Written informed consent was obtained from all subjects.

The subjects were 144 healthy university or college students [105 females and 39 males, 21.3±3.4 years of age (mean±SD)] who were recruited voluntarily from February to December 2008. The sample size was justified by the reported minor allele frequencies in Japanese population [5], [7]–[9]. Genotyping of the mtDNA C5178A and A10398G polymorphisms was carried out by using the polymerase chain reaction (PCR)-restriction fragment length polymorphism (RFLP) method based on the previous reports [5], [9]. The mitochondrial genomic DNA was extracted from buccal mucosal cells by using buccal cell DNA extraction kit, BuccalQuick™ and specially designed foam swab, Easy-Swab™ (TrimGen, Maryland, USA). The PCR amplifications were performed using DNA amplification kit, AccuPower® PCR PreMix (BIONEER, Daejeon, Korea) with the following primers: 5′-ATCCATCATAGCAGGCAGTT-3′ and 5′-GAGTAGATTAGGCGTAGGTA -3′ for the mtDNA C5178A polymorphism, 5′-TTATGTCATCCCTCTTATTAA-3′ and 5′-GTTTAAACTATATGCCAATTCGG-3′ for the mtDNA A10398G polymorphism. The PCR conditions were: 30 cycles of denaturation at 94°C for 30 s, annealing at 51°C for 30 s, and extension at 72°C for 90 s for C5178A, 35 cycles of 94°C for 30 s, 55°C for 30 s, and 72°C for 60 s for A10398G. The PCR products of the C5178A and A10398G sites were digested at 37°C overnight by *Alu* I and *Bgl* I (New England Biolabs, Massachusetts, USA), respectively. The 417 base pairs (bp) product was cut into three fragments of 188, 186, and 43 bp for the 5178C polymorphism, whereas it was split into two fragments of 374 and 43 bp for the 5178A polymorphism. The 101 bp product was digested into two fragments of 83 and 18 bp when the base at 10398 position was G. The electrophoresis was carried out by using buffer-free electrophoresis system, E-Gel® iBase Power System with ethidium bromide stained precast agarose gel, E-Gel® 4% (Invitrogen, California, USA). The fragments were visualized by ultraviolet transilluminator.

Temperament was assessed by means of the University Personality Inventory (UPI), which is a questionnaire developed by the Japan University Health Association that evaluates the mental health of university students [12]. The UPI consists of 60 items and is designed to evaluate four types of temperament (physical complaint, depression, anxiety, and obsession). For statistical analyses, Kruskal-Wallis test, and Mann-Whitney U-test were performed using SPSS 11.0 for Windows (SPSS, Tokyo, Japan). A p-value of <0.05 was considered significant.
